# Incipient Insanity

**Published:** 1923-06

**Authors:** 


THE
### HOSPITAL and HEALTH
REVIEW

### INCIPIENT INSANITY.

THERE are in this country at the present time 270,000 people who are either certified lunatics or are feeble-minded and consequently predisposed to insanity. Every year, on the average, 22,000 fresh cases of "lunacy," more or less accurately so called, are added to the terrible total, and the number who are discharged as cured is negligible. Thus the Mental Treatment Bill which has been brought in by the Ministry of Health, is clearly long overdue. The measure may suffer from defects of detail—most legislation does; but discussion and experience should remove most of them, and in the meantime the Bill may be broadly accepted as a long step forward in our dealings with incipient insanity and in the classification of mental disorders. It is notorious that there are many persons at present kept in mental hospitals who are not, in the true sense of the word, insane, but they are insane enough, with the rough and ready methods which have hitherto prevailed, to justify their detention. Putting aside for the moment the technically feeble-minded, there has been no half-way house—a person was either insane or he was not. But there are many unhappy persons who hover upon the borderline, and no inconsiderable proportion are themselves aware of their danger. To certify them may be to commit an act of cruelty to the individual; not to do so is sometimes to endanger both them and the community.

Every disease is preventable, if we know how to prevent it, and early and accurate diagnosis is the surest means either of prevention or cure in early stages. It is within the experience of every doctor that he has been consulted too late—there are, indeed, few more saddening, we might say more heartrending, experiences to the man who realises what science could have done had its aid been invoked in time. The mental specialist is by no means exempt from these trials—they fall upon him far more frequently than is generally supposed. But the means of dealing with disorders of the brain in their earliest stages have been few, and the law which provides for certification and confinement has lent no a.di Now we may fairly hope for better things. The chief purpose of the Mental Treatment Bill—in the form of a conversation with the Chairman of the Board of Control, we print on another page a clear exposition of its provisions—is to provide that a patient suffering from incipient mental disorder may be received for treatment without certification. The patient can be admitted only at his own request, which must be supported by the recommendation of two doctors, and the institution which he enters must be officially approved. He will stay for a maximum period of six months, with power to extend it for a further six months, and during either period he may leave by giving two days' notice. If, at the end of a year, he is not cured he can be certified. In this faculty of leaving at forty-eight hours' notice there is a weakness which is almost inevitable in a scheme which is intended to be purely voluntary in its operation. There are provisions for out-patient treatment and for after-care, and patients may be received in general hospitals. The foundation of the scheme rests upon the elimination of all suggestion of the asylum. The patient is to be given a real chance of recovery—a chance which will be greatly assisted by placing at his command all the resources of the skilled alienist.

Such a measure as this is necessarily experimental; but it is an experiment based upon assured facts. Notwithstanding the difficulties which have hitherto surrounded early treatment, enough has been accomplished to afford sound promise that a far larger measure of success will attend these efforts when they are co-ordinated and pursued in entirely favourable circumstances with all the help that science can give. That so few certified lunatics ever emerge is less surprising than that there are even occasional cures. When we have called an asylum a "mental hospital" we have done little more than soften a wounding phraseology. The surroundings remain unaltered; the atmosphere of insanity still envelops patients who, in a more hopeful environment would enjoy real opportunities of cure. It is to avoid the stigma of lunacy, to maintain hope, to help the patient to help himself, to stimulate his clouded faculties, to make his life interesting rather than to deaden it with monotony that the Mental Treatment Bill has been devised. It is not to be pretended that it will solve one of the most poignant problems of modern life. But, well administered and generously interpreted, it should help materially towards its solution. The test will be whether, allowance being made for the growth of population, it diminishes the number of certifications and restores to happiness and usefulness a substantial proportion of those who take advantage of the facilities the Bill provides. Signal as are the triumphs of preventive medicine in the strictly physical sense, we are not entitled to despair of their being rivalled in time by the triumphs of those who minister to the mind diseased.

